# Effects of Early Transplantation of the Faecal Microbiota from Tibetan Pigs on the Gut Development of DSS-Challenged Piglets

**DOI:** 10.1155/2021/9823969

**Published:** 2021-01-19

**Authors:** H. Diao, Y. Xiao, H. L. Yan, B. Yu, J. He, P. Zheng, J. Yu, X. B. Mao, D. W. Chen

**Affiliations:** ^1^Institute of Animal Nutrition, Sichuan Agricultural University, Key Laboratory for Animal Disease-Resistance Nutrition of China Ministry of Education, No. 46 Xinkang Road, Ya'an, Sichuan 625014, China; ^2^Animal Breeding and Genetics Key Laboratory of Sichuan Province, Sichuan Academy of Animal Science, No. 7 Niusha Road, Chengdu, Sichuan 610066, China

## Abstract

The present study was conducted to investigate the effects of early transplantation of the faecal microbiota from Tibetan pigs on the gut development of dextran sulphate sodium- (DSS-) challenged piglets. In total, 24 3-day-old DLY piglets were divided into four groups (*n* = 6 per group); a 2 × 2 factorial arrangement was used, which included faecal microbiota transplantation (FMT) (from Tibetan pigs) and DSS challenge. The whole trial lasted for 55 days. DSS infusion increased the intestinal density, serum diamine oxidase (DAO) activity, and colonic *Escherichia coli* count (*P* < 0.05), and decreased the *Lactobacillus* spp. count and mRNA abundances of epidermal growth factor (EGF), glucagon-like peptide-2 (GLP-2), insulin-like growth factor 1 (IGF-1), occludin, mucin 2 (MUC2), regeneration protein III*γ* (RegIII*γ*), and interleukin-10 (IL-10) in the colon (*P* < 0.05). FMT increased the *Lactobacillus* spp. count and mRNA abundances of GLP-2, RegIII*γ*, and IL-10 in the colon (*P* < 0.05), and decreased the intestinal density, serum DAO activity, and colonic *E. coli* number (*P* < 0.05). In addition, in DSS-challenged piglets, FMT decreased the disease activity index (*P* < 0.05) and attenuated the effect of DSS challenge on the intestinal density, serum DAO activity, and colonic *E. coli* number (*P* < 0.05). These data indicated that the faecal microbiota from Tibetan pigs could attenuate the negative effect of DSS challenge on the gut development of piglets.

## 1. Introduction

Over thousands of years of evolution, hosts and bacteria have developed beneficial relationships, creating a mutually beneficial symbiotic environment [1]. The microbiota contribute to many physiological processes in hosts. In turn, hosts provide a basic developmental environment for microorganisms [2, 3]. Recently, many studies in the biomedical field have shown that the intestinal microbiota are closely related to host health; these microorganisms can affect the digestion, absorption, and metabolism of nutrients, and regulate the physiological functions and the occurrence and development of diseases in hosts [4, 5].

The overall balance of gut microbial communities is important to ensure homeostasis in the intestinal mucosa [6, 7]. The comparison between traditional and sterile animals has revealed the vital function of the intestinal microbiota in the development of the gastrointestinal tract profile, such as villus thickness, Peyer's patch maturity, and the numbers of isolated lymphoid follicles [8–11]. The gut microbiota can be used as targets for regulating metabolism and treating intestinal diseases in hosts [12]. Transplanting faecal materials from healthy individuals into patients with different diseases, such as inflammatory bowel disease, *Clostridium difficile* infection, metabolic diseases, and immune disorders, can be beneficial for the treatment of these diseases [13–16]. In animal production, the study of the relationship between the microbiota and host is still in its infancy.

We previously found huge differences in the gut microbiota composition among pig breeds [17]. Moreover, we found that transplantation of the faecal microbiota from Rongchang and Yorkshire pigs disrupted the normal microbial balance in the intestinal tract of suckling piglets, which was not conducive to the normal development of the intestinal tract [18]. In contrast, transplantation of the faecal microbiota from Tibetan pigs reduced diarrhoea and promoted absorption enzyme activities in piglets [18]. However, the potential anti-inflammatory value and application of the faecal microbiota from Tibetan pigs have been scarcely studied. Hence, the present study was conducted to investigate the protective effects of faecal microbiota transplantation (FMT) (from Tibetan pigs) on dextran sulphate sodium- (DSS-) challenged suckling piglets in order to provide some new insights into the role of FMT in colitis.

## 2. Materials and Methods

### 2.1. Animals, Management, and Diets

Five Tibetan pigs (aged 12 weeks) were used in the present study as faecal donors. All the pigs were provided by a reservation farm and separately housed in individual metabolic cages in an environmentally controlled room for 8 weeks until sacrifice. The pigs were allowed ad libitum access to water and food. All the pigs received no antibiotics or probiotics during the latest 8-week period according to the standard for donor identification [19].

In total, 24 DLY suckling piglets (2.08 ± 0.12 kg) were separated from their mothers at 48 h after birth, and were then fed with artificial milk for 24 h. As a 2 × 2 factorial arrangement, all the piglets were randomly allotted to groups (*n* = 6 per group) matched for body weight and gender. The factors were FMT (piglets who received FMT from Tibetan pigs and those who did not) and challenge status (DSS-challenged and nonchallenged piglets). The four treatment groups were as follows: (1) the control (CTL) group, receiving only sterile PBS; (2) the CTL-T group, receiving FMT from Tibetan pigs; (3) the CTL-D group, receiving sterile PBS and DSS; and (4) the CTL-T-D group, receiving FMT from Tibetan pigs and DSS. All the piglets were artificially fed with milk substitutes in the first 15 days and then given solid feed in a gradual manner. The piglets in each group were separately housed in four environmentally controlled rooms and given the same diet throughout the process. The dietary compositions for each period are shown in Tables S1–S3. The entire trial lasted for 55 days.

### 2.2. Faecal Microbiota Transplantation

Fresh faecal samples were collected from five Tibetan pigs after 12 h of fasting and thoroughly mixed. Stool suspensions were prepared using a previously described method [17, 20]. In brief, 1 : 9 (*w*/*v*) sterile saline was added to the mixed fresh faeces, following which the suspension was mixed and passed through stainless steel laboratory sieves (2.0, 1.0, and 0.5 mm, respectively). The piglets in the CTL-T and CTL-T-D groups were intragastrically infused with 10 mL faecal suspension daily for days 1–3 and every 2 days for days 4–15 and with 20 mL faecal suspension every 5 days for days 16–46.

### 2.3. DSS Administration

Experimental colitis was induced by the intragastric administration of DSS (MW: 36000-50000, MP Biomedicals, USA) according to a previously described method [21]. In brief, following 12 h fasting, the piglets in the CTL-D and CTL-T-D groups were intragastrically infused with 200 mL DSS solution (4%) on day 51 and then with 100 mL DSS solution (4%) daily for days 52–55. The nonchallenged piglets (CTL and CTL-T groups) were infused with an equal volume of sterile saline.

### 2.4. Sample Collection

On day 56, following 12 h fasting, 10 mL blood samples were collected from the precaval vein of each piglet, and the serum was isolated by centrifugation at 3,000 r/min for 10 min. Following this, all the piglets were sacrificed by the injection of Zoletil 50 (BLESS Biotech, Beijing) at a dose of 10 mg/kg body weight and jugular exsanguinations. The length and weight of the intestine were measured after opening the abdomen. Sections of the jejunum, ileum, and colon were obtained, fixed in 10% buffered neutral formalin and embedded in paraffin for histological examination. Samples of the jejunum, ileum, and colon were immediately stored at -80°C for analysing the antioxidant capacity and mRNA expression levels of some genes. Digesta samples of the cecum and colon were immediately isolated and stored at -80°C for analysing the microbiota composition and metabolites.

### 2.5. Disease Activity Index (DAI)

DAI was assessed according to the diarrhoea score, faecal occult blood index, and body weight change rate on a daily basis after DSS challenge; it was calculated by modifying a mixed clinical score described previously [22]. Each day during the challenge, the body weight of each piglet was measured, the diarrhoea score for each piglet was visually assessed according to the scoring system described by Hart and Dobb in 1988 [23], and the faecal occult blood index for each piglet was measured using faecal occult blood test paper strips (by the Colloidal Gold Method, W.H.P.M. Biotech, Beijing).

### 2.6. Histology of Intestine

Next, 1 cm long samples of the jejunum, ileum, and colon were fixed in 10% formaldehyde solution, dehydrated, and embedded in paraffin wax. The preserved samples were prepared after cutting, installing, and staining with haematoxylin and eosin and/or periodic acid-Schiff and Alcian blue. In total, 10 well-orientated sections of villus-crypt units in the jejunum and ileum were randomly selected, and the villus height and crypt depth were measured by using a light microscope (Olympus, Tokyo, Japan) and a digital microscope camera (Olympus Optical Company, Guangzhou, China). In addition, the number of goblet cells in the ileum and colon was counted using a previously described method [24].

### 2.7. Lipopolysaccharide (LPS) and Diamine Oxidase (DAO) Concentrations in the Serum

A porcine-specific ELISA kit (R&D System, Minneapolis, MN) and a microplate reader (BioTek Instruments, Winooski, VT) were used to quantify the serum LPS concentration, in accordance with the manufacturer's protocol. The serum DAO concentration was measured using a commercial kit produced by Nanjing Jiancheng Bioengineering Institute (Jiangsu, China), and quantified by using a UV-vis spectrophotometer (UV1100, Shanghai, China).

### 2.8. Antioxidant Capacity and Glucagon-Like Peptide-2 (GLP-2) Level in the Jejunum and Colon

Supernatants of the jejunal and colonic samples were obtained according to the previously described methods [25]. In brief, the jejunum and colon were homogenised with sterile saline (*m*/*v* = 1/9) and centrifuged at 500 × g for 15 min at 4°C. The supernatant was used for assessing the total protein concentration, total antioxidant capacity (T-AOC), malondialdehyde (MDA) concentration and superoxide dismutase (SOD) activity using commercial kits produced by Nanjing Jiancheng Bioengineering Institute (Jiangsu, China) in accordance with the manufacturer's protocol. The GLP-2 concentrations in the jejunum and colon were assessed using Pig Enzyme-Linked Immunosorbent Assay Kits (R&D System, Minneapolis, MN), and quantified using a BioTek Synergy HT Microplate Reader (BioTek Instruments, Winooski, VT).

### 2.9. Total RNA Extraction, Reverse Transcription Reaction, and Real-Time Quantitative PCR

Expression levels of targeted genes, including those encoding insulin-like growth factor 1 (IGF-1), GLP-2, epidermal growth factor (EGF), insulin-like growth factor 1 receptor (IGF-1R), angiogenin 4 (ANG4), mucin 1 (MUC1), regeneration protein III*γ* (RegIII*γ*), mucin 2 (MUC2), zonula occludens 1 (ZO-1), occludin, interleukin-10 (IL-10), and interleukin-1*β* (IL-1*β*) in the jejunum, ileum, and colon, were analysed by real-time PCR using the CFX96 Real-Time PCR Detection System (Bio-Rad, Richmond, CA) according to a previously described method [26]. In brief, total RNA was isolated from the frozen jejunum, ileum, and colon using the TRIzol Reagent (Takara Bio Inc., Dalian, China) in accordance with the manufacturer's protocols. Following this, RNA samples were reverse transcribed into complementary DNA (cDNA) using the PrimeScript™ RT reagent kit (Takara Bio Inc., Dalian, China). Finally, a 10 *μ*L quantitative fluorescent PCR reaction volume was used, which consisted of 1 *μ*L cDNA, 0.5 *μ*L upstream and downstream primers, 5 *μ*L SYBR Premix Ex Taq™, and 3 *μ*L RNase-free H_2_O. The PCR cycle conditions were as follows: 30 s at 95°C, 10 s at 95°C, and 25 s at 60°C for a total of 40 cycles. The primers shown in Table S4 were commercially synthesised by Invitrogen (Shanghai, China). The expression level of each gene in the tissues was calculated using *β*-actin as the reference gene.

### 2.10. Microbial Population Determination

The caecal and colonic digesta samples were used to extract bacterial DNA using Stool DNA kits (Omega Bio-tek, Doraville, CA). To quantify the microbial population, the primers and fluorescent oligonucleotide probes (Table S5) for total bacteria, *Bacillus* spp., *Lactobacillus* spp., *Escherichia coli*, and *Bifidobacterium* spp. were obtained according to the previously described methods [27, 28]. Quantitative real-time PCR was performed using the CFX96 Real-Time PCR Detection System (Bio-Rad, CA, USA). A 25 *μ*L quantitative fluorescent PCR reaction volume was used for counting the total bacteria; it consisted of 1 *μ*L DNA, 1 *μ*L each of upstream and downstream primers, 12.5 *μ*L SYBR Premix Ex Taq™, and 9.5 *μ*L ddH_2_O. For counting the other bacteria, a 20 *μ*L PCR reaction volume was used; it consisted of 1 *μ*L DNA, 1 *μ*L each of upstream and downstream primers, 0.3 *μ*L probe, 1 *μ*L probe enhancer solution, 8 *μ*L RealMasterMix, and 7.7 *μ*L ddH_2_O. The PCR conditions and computing method were consistent with those reported by Qi et al. [27].

### 2.11. Short-Chain Fatty Acids (SCFAs)

Frozen colonic digesta samples were used to measure SCFA levels using the Varian CP-3800 gas chromatographic system (Palo Alto, CA, USA), as described previously [29]. In brief, the colonic digesta were homogenised with distilled water (*m*/*v* = 1/1) and centrifuged at 500 × g for 10 min. Then, 2 mL of supernatant was collected and centrifuged at 12,000 × g for 10 min. Following this, 0.2 mL of 25% metaphosphoric acid was added to 1 mL of the supernatant, kept for 30 min, and centrifuged at 12,000 × g for 10 min. An equal volume of methanol was added to the supernatant and centrifuged at 12,000 × g for 10 min. Finally, the supernatant was collected and stored at -20°C for measuring the acetic acid, propionic acid, and butyric acid levels.

### 2.12. Statistical Analysis

The results are expressed as the means and SEM. Statistical analysis was performed by Student's *t*-test or two-way ANOVA using the statistical software SAS 8.2 (SAS Inst. Inc., NC); each piglet was the statistical unit. Significance was accepted at *P* < 0.05, while *P* < 0.10 was considered a tendency.

## 3. Results

### 3.1. DAI

**Figure 1:**
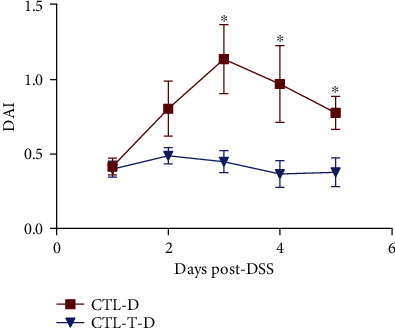
Effect of gut microbiota intervention on disease activity index (DAI) of dextran sulphate sodium- (DSS-) challenged piglets. CTL-D: piglets infused with sterile PBS and DSS. CTL-T-D: piglets infused with the faecal microbiota from Tibetan pigs and DSS. ^∗^*P* < 0.05.

On days 3–5, FMT (from Tibetan pigs) significantly decreased the DAI in the DSS-challenged piglets (*P* < 0.05, Figure 1).

### 3.2. Intestinal Index

The effects of FMT and DSS challenge on the intestinal index of the piglets are shown in Table 1. DSS challenge increased the large intestinal density of the piglets (*P* < 0.05). In the piglets, FMT decreased the large intestinal density and increased the length of the small intestine and whole intestine (*P* < 0.05). Moreover, FMT attenuated the effect of DSS challenge on the density of the large intestine (*P* < 0.05) and whole intestine (*P* = 0.094).

DSS-: infused with sterile saline; DSS+: infused with DSS; Micro-: infused with sterile saline; Micro+: infused with the faecal microbiota from Tibetan pigs; SI: small intestine; LI: large intestine; I: whole intestine. ^a-b^Within a row, means without a common superscript differ (*P* < 0.05).

### 3.3. Intestinal Morphology and Number of Goblet Cells

The villus height and crypt depth in the jejunum and ileum and the number of goblet cells in the ileum and colon are indicated in Table 2. In the piglets, DSS challenge increased the villus height and crypt depth in the jejunum and ileum and decreased the villus height : crypt depth ratio in the jejunum and the number of goblet cells in the colon (*P* < 0.05). FMT decreased the villus height and crypt depth in the jejunum and ileum of the piglets (*P* < 0.05). Moreover, FMT relieved the effect of DSS challenge on the villus height and crypt depth in the jejunum and ileum of the piglets (*P* < 0.05).

DSS-: infused with sterile saline; DSS+: infused with DSS; Micro-: infused with sterile saline; Micro+: infused with the faecal microbiota from Tibetan pigs. ^a-b^Within a row, means without a common superscript differ (*P* < 0.05).

### 3.4. Relative mRNA Expression Levels of Intestinal Development-Related Genes and GLP-2 Concentrations

As shown in Table 3, DSS challenge decreased the mRNA expression of ANG4 in the jejunum (*P* < 0.05). It also decreased the mRNA expressions levels of EGF, GLP-2, ANG4, and IGF-1 in the colon (*P* < 0.05), and the GLP-2 concentrations in the jejunum (*P* < 0.05) and colon (*P* = 0.099) of the piglets. FMT increased the mRNA expression level of ANG4 in the jejunum (*P* < 0.05) and the mRNA expression levels and concentrations of GLP-2 in the jejunum and colon of the piglets (*P* < 0.05). However, no significant interaction effects were noted with regard to the mRNA expression levels of intestinal development-related genes and GLP-2 concentrations between DSS administration and FMT (*P* > 0.05).

DSS-: infused with sterile saline; DSS+: infused with DSS; Micro-: infused with sterile saline; Micro+: infused with the faecal microbiota from Tibetan pigs; EGF: epidermal growth factor; IGF-1: insulin-like growth factor 1; GLP-2: glucagon-like peptide 2; IGF-1R: insulin-like growth factor 1 receptor; ANG4: angiogenin 4. ^a-b^Within a row, means without a common superscript differ (*P* < 0.05).

### 3.5. Intestinal Antioxidant Capacity

The antioxidant capacity in the jejunum and colon of the piglets is summarised in Table 4. DSS challenge increased the MDA concentrations in the jejunum and colon (*P* < 0.05) and decreased the T-AOC capacity and SOD activity in the colon of the piglets (*P* < 0.05). FMT increased the T-AOC capacity and SOD activity in the jejunum (*P* < 0.05) and decreased the MDA concentration in the colon of the piglets (*P* < 0.05). In addition, FMT attenuated the effect of DSS challenge on the MDA concentration and SOD activity in the colon of the piglets (*P* < 0.05).

DSS-: infused with sterile saline; DSS+: infused with DSS; Micro-: infused with sterile saline; Micro+: infused with the faecal microbiota from Tibetan pigs; T-AOC: total antioxidant capacity; MDA: malondialdehyde; SOD: superoxide dismutase. ^a-b^Within a row, means without a common superscript differ (*P* < 0.05).

### 3.6. Intestinal Barrier Function

As shown in Table 5, DSS challenge increased the serum DAO activity (*P* < 0.05) and enhanced the mRNA expression level of IL-1*β* in the colon (*P* < 0.05). Moreover, it decreased the mRNA expression levels of MUC1 and MUC2 in the jejunum (*P* < 0.05) and the mRNA expression levels of occludin, MUC2, RegIII*γ*, and IL-10 in the colon of the piglets (*P* < 0.05). FMT decreased the serum DAO activity (*P* < 0.05), downregulated the mRNA expression level of IL-1*β* in the colon (*P* < 0.05), and upregulated the mRNA expression levels of RegIII*γ* and IL-10 in the colon of the piglets (*P* < 0.05). FMT also attenuated the effect of DSS challenge on the serum DAO activity (*P* < 0.05) and the mRNA expression level of RegIII*γ* in the colon (*P* = 0.082) of the piglets.

DSS-: infused with sterile saline; DSS+: infused with DSS; Micro-: infused with sterile saline; Micro+: infused with the faecal microbiota from Tibetan pigs; ZO-1: zonula occludens 1; MUC1: mucin 1; MUC2: mucin 2; REGIII*γ*: regeneration protein III*γ*; IL-10: interleukin-10; IL-1*β*: interleukin-1*β*; LPS: lipopolysaccharide; DAO: diamine oxidase. ^a-b^Within a row, means without a common superscript differ (*P* < 0.05).

As shown in Tables 6 and 7, DSS challenge increased the *E. coli* count and the propionic acid and total SCFA levels (*P* < 0.05) and decreased the *Lactobacillus* spp. count and the butyric acid level (*P* < 0.05) in the colonic digesta of the piglets. FMT increased the *Lactobacillus* spp. and *Bacillus* spp. counts in the caecal digesta and the total bacteria and *Lactobacillus* spp. counts in the colonic digesta (*P* < 0.05). It decreased the *E. coli* counts in the caecal and colonic digesta and the acetic acid, propionic acid, and total SCFA levels in the colonic digesta of the piglets (*P* < 0.05). Moreover, FMT attenuated the effect of DSS challenge on the *E. coli* count (*P* = 0.072) and the acetic acid (*P* < 0.05), propionic acid (*P* < 0.05), and total SCFA (*P* < 0.05) levels in the colonic digesta of the piglets.

DSS-: infused with sterile saline; DSS+: infused with DSS; Micro-: infused with sterile saline; Micro+: infused with the faecal microbiota from Tibetan pigs. ^a-b^Within a row, means without a common superscript differ (*P* < 0.05).

DSS-: infused with sterile saline; DSS+: infused with DSS; Micro-: infused with sterile saline; Micro+: infused with the faecal microbiota from Tibetan pigs. ^a-b^Within a row, means without a common superscript differ (*P* < 0.05).

## 4. Discussion

The number of intestinal microbes is dynamically balanced, and an imbalance can cause an inflammatory response by the immune system [30]. Colitis is a common gastrointestinal dysfunction disease, and it is clinically manifested as diarrhoea and bloody stools, which are associated with an abnormal immune response induced by intestinal microbiome disorders [31]. In the present study, in order to explore the potential benefits of FMT from Tibetan pigs in an inflammatory model and its application value in animal husbandry production, DSS was orally infused in piglets. DSS challenge increased the DAI, visible bloody stools, and diarrhoea in the piglets, particularly in those without FMT. In addition, DSS infusion impaired the gut histology, development, antioxidant capacity, and barrier function. These results were consistent with those of previous studies in mice, indicating that our colitis model was successful [32–34].

In the present study, we also found that FMT from Tibetan pigs significantly reduced the DAI in the DSS-challenged piglets, indicating that early FMT from Tibetan pigs can alleviate the clinical symptoms induced by DSS infusion. *Lactobacillus acidophilus* can reduce the increase in the DAI and colonic histopathology scores triggered by DSS challenge in mice and has a therapeutic effect on acute ulcerative colitis [35]. Similarly, colitis induced by DSS challenge can be relieved by *Bifidobacterium* treatment in the mice with a decreasing diarrhoea score and colonic hyperaemia [36]. In our previous study, 16S rRNA gene sequencing demonstrated that the gut microbiota profile differs among three pig breeds (Yorkshire pigs, Tibetan pigs, and Rongchang pigs), with higher *Lactobacillus* spp. and *Parabacteroides* spp. counts being observed in Tibetan pigs [17]. *Lactobacillus* and *Parabacteroides* are two genera considered to be positively correlated with the cure of colitis [37, 38]. The present study also revealed that the piglets who received the stool suspensions from Tibetan pigs had higher copies of *Lactobacillus* spp. in their colonic digesta, which may be an important reason why DSS-induced colitis is relieved by FMT from Tibetan pigs.

As DSS primarily causes colon damage, most studies have focused on the large intestine, with few studies focusing on the small intestine. In the present study, DSS challenge increased the large intestinal density, villus height, and crypt depth in the jejunum and ileum and decreased the villus height : crypt depth ratio in the jejunum and the mRNA expression levels of EGF, GLP-2, ANG4, and IGF-1 in the colon of the piglets. Thus, DSS challenge not only impaired the development of the colon but also negatively affected the jejunum and ileum of the piglets. We also found that FMT attenuated the effect of DSS challenge on the large intestinal density, villus height, and crypt depth in the jejunum and the GLP-2 concentrations in the jejunum and colon of the piglets. It is well known that EGF and IGF-1 are the main regulators of intestinal cell proliferation [39, 40]. ANG4 is a Paneth cell granule protein that plays an important role in shaping intestinal angiogenesis [41]. GLP-2, a specific growth regulator of intestinal epithelial cells, is a hormone that is mainly synthesised and secreted by intestinal endocrine cells. It stimulates the intestinal blood flow and intestinal cell proliferation and thus promotes the growth of the intestinal mucosa and improves nutrient absorption [42–44]. Changes in the intestinal microbiota composition would affect endogenous GLP-2 production [45]. Therefore, early FMT from Tibetan pigs can attenuate the negative effect of DSS infusion on the intestinal development of piglets, which may be associated with the GLP-2 production.

The intestinal barrier function plays an important role in gut health; the effects of DSS challenge and FMT on gut health are mainly reflected in the intestinal barrier function. In the inner barrier of the intestinal mucosa, the tight junction is mainly composed of the peripheral membrane protein (ZO family) and transmembrane protein (occludin and claudin families), which participate in the formation of the intestinal mucosal barrier and play a decisive role in the intestinal barrier [46]. The integrity of tight junctions affects the intestinal inflammatory response [46]. DSS challenge could increase the intestinal permeability, decrease the mRNA and protein expression levels of occludin and claudin-1 in the colon, and destroy the inner barrier of the gut mucosa in mice [47, 48]. Mice colonised with *Lacticaseibacillus rhamnosus* have a low serum D-lactic acid level and a low susceptibility to colitis [49]. Similar results were observed in the present study: DSS challenge significantly increased the serum DAO activity and MDA concentrations in the jejunum and colon and decreased the T-AOC, SOD activity, and mRNA expression level of occludin in the colon of the piglets. FMT from Tibetan pigs significantly alleviated the effect of DSS challenge on the serum DAO activity and colonic MDA concentration of the piglets. The outer barrier of the intestinal mucosa consists of normal intestinal microbiota, a mucous layer, and secreted immunoglobulin A. It inhibits the intestinal adhesion and implantation of pathogenic bacteria [50]. Under normal conditions, the microbial count in the loose adhesive mucous layer is higher than that in the strong adhesive mucous layer in the colon of rats. However, the microbiota migrates from the loose adhesive mucous layer to the strong adhesive mucous layer after DSS challenge; this changes the structure and composition of the intestinal microbiota in the loose adhesive mucous layer and the strong adhesive mucous layer in the colon [51]. DSS-induced colitis can reduce the *Lactobacillus* spp. count and increase the *E. coli* count in the intestines. In addition, it can increase the plasma LPS concentration and the expression of proinflammatory factors (such as IL-6, IL-1*β*, IFN-*γ*, and IL-12) in the colon of rats/mice through Toll-like receptor 4 signals [52, 53]. A significant reduction in the colonic expression level of MUC2 has been observed in patients with enteritis [54]. MUC2-knockout mice have been found to be more sensitive to DSS-induced colitis and presented with severely damaged mucosa, indicating that MUC2 plays an indispensable role in the gut barrier function [55, 56]. In the present study, DSS challenge increased the *E. coli* count and propionic acid and total SCFA levels in the colonic digesta, while it decreased the numbers of goblet cells in the colon and the *Lactobacillus* spp. count and butyric acid levels in the colonic digesta; it also decreased the mRNA expression levels of MUC1 and MUC2 in the jejunum and the mRNA expression levels of MUC2, RegIII*γ*, and IL-10 in the colon. It is speculated that the increase in SCFA levels is a compensatory manifestation in the inflammatory model, which is abnormal accumulation and may cause the deepening of inflammation. Moreover, we found that FMT from the Tibetan pigs attenuated the effect of DSS challenge on *E. coli* count and propionic acid and total SCFA levels in the colonic digesta of piglets. Previous research has revealed that *Lactiplantibacillus plantarum* administration could enhance the ratio of *Firmicutes* : *Bacteroidetes* ratio and diversify the microbial species in the colon of mice [35] and prevent the migration of microorganisms from the loose adhesive mucous layer to the strong adhesive mucous layer in the colon of rats [51]. In addition, probiotic treatment or FMT can reduce the expression and secretion of inflammatory factors (such as IFN-*γ*, IL-12, TNF-*α*, IL-6, and IL-1*β*) to resist colitis by regulating STAT1, STAT4, or NF-*κ*B signals [57, 58]. As indicated above, it can be speculated that FMT from the Tibetan pigs can attenuate the damage caused by DSS challenge, which can be related to the protective function of the intestinal barrier.

## 5. Conclusions

**Figure 2:**
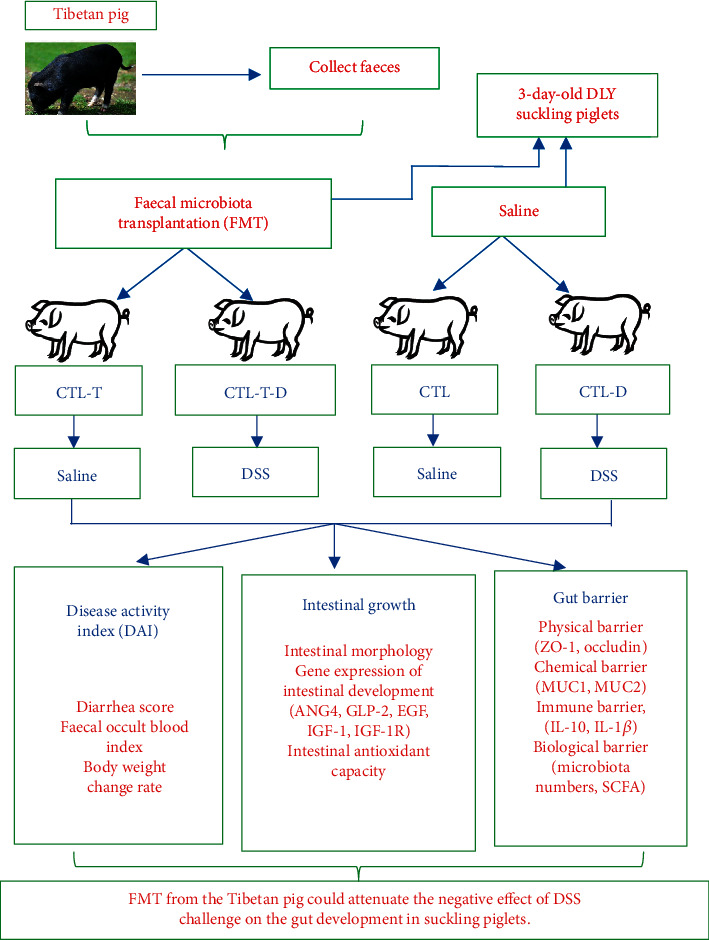
The overall frame diagram. CTL: receiving only sterile PBS. CTL-T: receiving the faecal microbiota from Tibetan pigs. CTL-D: receiving sterile PBS, followed by treatment with dextran sulphate sodium (DSS). CTL-T-D: receiving the faecal microbiota from Tibetan pigs, followed by treatment with DSS.

In summary, DSS infusion can damage the gut health of piglets. However, FMT from Tibetan pigs can attenuate the negative effect of DSS challenge on intestinal development by improving the gut barrier function of the piglets (Figure 2).

## Figures and Tables

**Table 1:** Effects of DSS challenge and FMT on the intestinal index in piglets.

Items	DSS-		DSS+		SEM	*P* value		
	Micro-	Micro+	Micro-	Micro+		Micro	DSS	Micro × DSS
Relative length of SI (cm/g)	5.257	5.498	5.003	5.777	0.231	0.040	0.957	0.264
Relative length of LI (cm/g)	1.195	1.208	1.092	1.217	0.056	0.234	0.409	0.333
Relative length of I (cm/g)	6.453	6.073	6.095	6.993	0.273	0.049	0.902	0.250
Relative density of SI (g/cm)	0.735	0.748	0.718	0.683	0.030	0.722	0.190	0.431
Relative density of LI (g/cm)	1.673^b^	1.688^b^	2.125^a^	1.707^b^	0.083	0.024	0.010	0.016
Relative density of I (g/cm)	0.908	0.918	0.972	0.858	0.035	0.156	0.963	0.094
Relative weight of SI (%)	3.862	4.058	3.598	3.958	0.186	0.150	0.341	0.666
Relative weight of LI (%)	2.003	2.005	2.315	2.060	0.115	0.284	0.126	0.277
Relative weight of I (%)	6.062	5.865	6.018	5.915	0.250	0.555	0.990	0.854

**Table 2:** Effects of DSS challenge and FMT on the intestinal morphology and number of goblet cells in the intestines of piglets.

Items	DSS−		DSS+		SEM	*P* value		
	Micro-	Micro+	Micro-	Micro+		Micro	DSS	Micro × DSS
*Jejunum*								
Villus height (*μ*m)	521.360^b^	546.800^b^	1168.700^a^	485.680^b^	70.195	<0.001	<0.001	<0.001
Crypt depth (*μ*m)	229.620^b^	210.290^b^	554.230^a^	218.530^b^	35.267	<0.001	<0.001	<0.001
Villus height : crypt depth	2.306	2.631	2.112	2.254	0.077	0.115	0.059	0.524
*Ileum*								
Villus height (*μ*m)	609.830^b^	563.870^b^	1161.670^a^	442.720^b^	69.900	<0.001	0.004	<0.001
Crypt depth (*μ*m)	244.020^b^	229.960^b^	528.700^a^	181.300^b^	33.556	<0.001	<0.001	<0.001
Villus height : crypt depth	2.615	2.464	2.195	2.497	0.084	0.655	0.259	0.190
*Goblet cells*								
Ileum	69.875	75.600	73.875	71.981	2.201	0.688	0.968	0.427
Colon	102.197^a^	100.656^a^	83.200^b^	74.200^b^	3.348	0.237	<0.001	0.397

**Table 3:** Effects of DSS challenge and FMT on the mRNA expression levels of intestinal development-related genes and GLP-2 concentrations in the intestines of piglets.

Items	DSS−		DSS+		SEM	*P* value		
	Micro-	Micro+	Micro-	Micro+		Micro	DSS	Micro × DSS
*Jejunum*								
EGF	1.000	1.071	0.913	0.911	0.052	0.749	0.263	0.740
GLP-2	1.000	1.346	0.775	1.161	0.087	0.034	0.740	0.903
ANG4	1.000^ab^	1.344^a^	0.624^b^	0.935^ab^	0.074	0.008	0.002	0.887
IGF-1	1.000	1.145	0.887	1.022	0.067	0.323	0.402	0.972
IGF-1R	1.000	1.085	0.979	0.984	0.036	0.553	0.426	0.601
*Ileum*								
EGF	1.000	1.190	0.911	0.979	0.047	0.192	0.129	0.465
GLP-2	1.000	1.178	1.161	1.000	0.037	0.107	0.107	0.390
ANG4	1.000	1.085	0.935	0.967	0.053	0.571	0.345	0.667
IGF-1	1.000	1.074	1.022	0.956	0.043	0.589	0.309	0.786
IGF-1R	1.000	1.019	0.984	1.002	0.026	0.375	0.393	0.586
*Colon*								
EGF	1.000	1.352	0.515	0.787	0.129	0.207	0.040	0.870
GLP-2	1.000^ab^	1.599^a^	0.348^b^	1.182^ab^	0.136	0.003	0.020	0.585
ANG4	1.000	1.081	0.700	0.607	0.071	0.963	0.005	0.491
IGF-1	1.000^ab^	1.215^a^	0.656^b^	0.701^b^	0.068	0.228	0.001	0.427
IGF-1R	1.000	1.137	0.902	0.993	0.081	0.509	0.485	0.896
*GLP-2 concentration (pmol/gprot)*								
Jejunum	3.052^ab^	3.260^a^	2.856^b^	2.936^b^	0.046	0.061	0.002	0.386
Colon	2.721^ab^	3.594^a^	2.164^b^	3.288^ab^	0.269	0.002	0.099	0.724

**Table 4:** Effects of DSS challenge and FMT on the colonic antioxidant capacity of piglets.

Items	DSS-		DSS+		SEM	*P* value		
	Micro-	Micro+	Micro-	Micro+		Micro	DSS	Micro × DSS
*Jejunum*								
MDA (nmol/mg protein)	0.863	0.930	1.127	1.029	0.038	0.818	0.015	0.242
T-AOC (U/mg protein)	0.259	0.296	0.214	0.288	0.013	0.029	0.266	0.446
SOD (U/mg protein)	88.654	98.499	77.264	92.747	16.146	0.053	0.179	0.652
*Colon*								
MDA (nmol/mg protein)	1.339^a^	1.016^b^	1.583^a^	1.025^b^	0.056	<0.001	0.014	0.021
T-AOC (U/mg protein)	0.328^ab^	0.422^a^	0.242^b^	0.387^a^	0.061	0.554	0.077	0.126
SOD (U/mg protein)	133.097	126.493	110.154	124.733	6.044	0.745	0.032	0.016

**Table 5:** Effects of DSS challenge and FMT on the serum DOA activity and LPS concentration and on the mRNA expression levels of intestinal barrier-related genes in the intestines of piglets.

	DSS-		DSS+		SEM	*P* value		
	Micro-	Micro+	Micro-	Micro+		Micro	DSS	Micro × DSS
*Jejunum*								
Occludin	1.000	0.946	0.945	0.859	0.034	0.327	0.319	0.826
ZO-1	1.000	1.024	0.992	0.933	0.035	0.819	0.508	0.580
MUC1	1.000^ab^	1.186^a^	0.316^b^	0.364^b^	0.088	0.159	<0.001	0.397
MUC2	1.000	1.010	0.545	0.570	0.083	0.908	0.007	0.962
RegIII*γ*	1.000	1.167	0.804	0.974	0.067	0.215	0.155	0.991
*Ileum*								
Occludin	1.000	0.965	0.929	0.867	0.055	0.680	0.475	0.906
ZO-1	1.000	1.011	0.910	0.962	0.042	0.728	0.441	0.819
MUC1	1.000	1.119	1.119	0.898	0.049	0.723	0.176	0.402
MUC2	1.000	0.980	0.962	1.049	0.059	0.794	0.903	0.677
RegIII*γ*	1.000	1.023	0.940	0.919	0.053	0.992	0.468	0.845
*Colon*								
Occludin	1.000^a^	0.890^a^	0.623^b^	0.609^b^	0.047	0.364	<0.001	0.484
ZO-1	1.000	0.915	0.747	0.766	0.064	0.803	0.137	0.690
MUC1	1.000	1.060	0.844	1.025	0.044	0.180	0.285	0.495
MUC2	1.000^ab^	1.244^a^	0.717^b^	0.801^b^	0.114	0.165	0.005	0.490
RegIII*γ*	1.000^a^	1.030^a^	0.265^c^	0.555^b^	0.074	0.035	<0.001	0.082
IL-1*β*	1.000^b^	0.839^b^	1.631^a^	1.182^b^	0.074	0.003	<0.001	0.120
IL-10	1.000^c^	1.531^ab^	1.228^bc^	1.739^a^	0.080	0.001	0.074	0.931
*Serum*								
DAO (U/L)	10.418	9.987	11.492	10.476	0.140	<0.001	<0.001	0.099
LPS (ng/mL)	68.155	67.322	70.929	69.472	1.208	0.654	0.340	0.903

**Table 6:** Effects of DSS challenge and FMT on the caecal and colonic *E. coli*, *Lactobacillus* spp., *Bifidobacterium* spp., *Bacillus* spp., and total bacterial counts in caecal and colonic digesta of piglets (log(copies/g)).

Items	DSS-		DSS+		SEM	*P* value		
	Micro-	Micro+	Micro-	Micro+		Micro	DSS	Micro × DSS
*Cecum*								
Total bacteria	11.503	11.422	11.481	11.422	0.028	0.150	0.665	0.958
*Bacillus* spp.	9.777	9.898	9.798	9.943	0.026	0.011	0.493	0.805
*Lactobacillus* spp.	8.345^b^	8.965^a^	8.145^b^	8.707^ab^	0.146	0.001	0.150	0.851
*E. coli*	8.574^ab^	8.157^b^	9.078^a^	8.088^b^	0.147	0.001	0.252	0.134
*Bifidobacterium* spp.	8.021	8.006	7.898	8.007	0.037	0.549	0.440	0.434
*Colon*								
Total bacteria	11.532^ab^	11.670^a^	11.423^b^	11.736^a^	0.037	0.001	0.706	0.137
*Bacillus* spp.	9.933	10.066	9.836	9.939	0.037	0.113	0.131	0.833
*Lactobacillus* spp.	8.475^bc^	9.388^a^	7.878^c^	8.731^ab^	0.181	<0.001	0.003	0.875
*E. coli*	8.740^ab^	8.153^b^	9.507^a^	8.274^b^	0.131	<0.001	0.007	0.072
*Bifidobacterium* spp.	7.629	7.931	7.798	7.801	0.052	0.142	0.848	0.152

**Table 7:** Effects of DSS challenge and FMT on SCFAs levels in the colonic digesta of piglets (*μ*mol/g).

Items	DSS-		DSS+		SEM	*P* value		
	Micro-	Micro+	Micro-	Micro+		Micro	DSS	Micro × DSS
Acetic acid	55.828^b^	53.410^b^	63.297^a^	51.716^b^	1.214	<0.001	0.103	0.014
Propionic acid	31.384^b^	29.340^b^	42.086^a^	27.223^b^	1.310	<0.001	0.001	<0.001
Butyric acid	16.973	16.544	15.132	15.524	0.335	0.978	0.036	0.527
Total volatile fatty acid	104.185^b^	99.294^bc^	120.514^a^	94.463^c^	2.353	<0.001	0.033	<0.001

## Data Availability

All data generated or analysed during this study are available from the corresponding authors on reasonable request.

## Supplementary Materials

Supplementary Materials Table S1: composition and nutrient level of artificial milk from days 1 to 15. Table S2: composition and nutrient level of experimental diets from days 16 to 28. Table S3: composition and nutrient level of experimental diets from days 29 to 56. Table S4: primer sequences and annealing temperature. Table S5: primes and probes for real-time PCR analysis of bacteria.
