# Supplementary material for: Effects of Early Transplantation of the Faecal Microbiota from Tibetan Pigs on the Gut Development of DSS-Challenged Piglets
Source: Biomed Res Int. 2021 Jan 19;2021:9823969. doi: 10.1155/2021/9823969 (PMC7837763; doi:10.1155/2021/9823969)
Supplement: Supplementary Materials — Table S1: composition and nutrient level of artificial milk from days 1 to 15. Table S2: composition and nutrient level of experimental diets from days 16 to 28. Table S3: composition and nutrient level of experimental diets from days 29 to 56. Table S4: primer sequences and annealing temperature. Table S5: primes and probes for real-time PCR analysis of bacteria. [file 9823969.f1.doc]

Table S1 Composition and nutrient level of artificial milk from days 1–15 (air dry basis %)

| Ingredient | Content | Calculated Composition | Nutrient content |
| --- | --- | --- | --- |
| [Whole](javascript:void(0);) [milk](javascript:void(0);) [powder](javascript:void(0);) | 58.00 | DE（Mcal/kg） | 4.29 |
| Whey protein concentrate | 25.00 | Crude protein | 27.26 |
| [Casein](javascript:void(0);) | 5.70 | Calcium | 0.87 |
| Coconut oil | 10.00 | Total phosphorus | 0.61 |
| Dicalcium phosphate | 0.10 | Available phosphorus | 0.61 |
| Chloride choline | 0.10 | Lys | 2.06 |
| Vitamin premix^1^ | 0.10 | Met | 0.88 |
| Mineral premix^2^ | 0.50 | Cys | 0.25 |
| Arg (99%) | 0.06 | Thr | 1.46 |
| *DL*-Met (99%) | 0.06 | Trp | 0.60 |
| *L-*Lys-HCl (78%) | 0.30 | Leu | 2.69 |
| Thr (98.5%) | 0.03 | Ile | 1.39 |
| Trp (98%) | 0.05 | Val | 1.54 |
| Total | 100.00 | Arg | 0.88 |

^1^The premix provides following per kg diet：VA11024 IU, VD_3_ 4500 IU, VE 48 mg, VK_3_6 mg, VB_2_ 12 mg, VB_6_ 6 mg, VB_12_ 48 μg, folic acid 2.4 mg, nicotinic acid 28 mg, biotin 300 μg, *D*-pantothenic acid 30 mg.

^2^The premix provides following per kg diet：Fe 100 mg, Cu 6 mg, Mn 4 mg, Zn 100 mg, I 0.14 mg, Se 0.3 mg.

Table S2 Composition and nutrient level of experimental diets from days 16–28 (air dry basis %)

| Ingredient | Content | Calculated Composition | Nutrient content |
| --- | --- | --- | --- |
| Corn | 13.06 | DE（MJ/kg） | 14.99 |
| Extruded corn | 26.51 | Crude protein | 21.87 |
| Extruded soybean meal | 9.00 | Crude fiber | 2.21 |
| Dehulled soybean meal | 2.00 | Lys | 1.68 |
| Fat powder | 1.00 | Trp | 0.29 |
| Sucrose | 2.00 | Thr | 1.07 |
| Glucose | 3.00 | Met+Cys | 0.99 |
| [Lactose](javascript:void(0);) | 7.00 | Calcium | 0.90 |
| [Whole](javascript:void(0);) [milk](javascript:void(0);) [powder](javascript:void(0);) | 4.50 | Total phosphorus | 0.70 |
| Whey Powder | 12.00 | Available phosphorus | 0.57 |
| Fish meal | 4.00 |  |  |
| Sprayed dried plasma protein | 6.50 |  |  |
| [Soy](javascript:void(0);) [protein](javascript:void(0);) [concentrate](javascript:void(0);) | 6.50 |  |  |
| *L-*Lys-HCl (78%) | 0.30 |  |  |
| *DL*-Met (99%) | 0.14 |  |  |
| Trp (98%) | 0.01 |  |  |
| Thr (98.5%) | 0.08 |  |  |
| Chloride choline | 0.10 |  |  |
| Dicalcium phosphate | 0.90 |  |  |
| Limestone | 0.87 |  |  |
| Nacl | 0.10 |  |  |
| Vitamin premix^1^ | 0.05 |  |  |
| Mineral premix^2^ | 0.40 |  |  |
| Total | 100.00 |  |  |

^1^The premix provides following per kg diet：VA 5512 IU, VD_3_2250 IU, VE 24 mg, VK_3_ 3 mg, VB_2_ 6 mg, VB_6_ 3 mg, VB_12_ 24 μg, folic acid 1.2 mg, nicotinic acid 14 mg, biotin 150 μg, *D*-pantothenic acid 15 mg.

^2^The premix provides following per kg diet：Fe 100 mg, Cu 6 mg, Mn 4 mg, Zn 100 mg, I 0.14 mg, Se 0.3 mg.

Table S3 Composition and nutrient level of experimental diets from days 29–56 (air dry basis %)

| Ingredient | Content | Calculated Composition | Nutrient content |
| --- | --- | --- | --- |
| Corn | 27.79 | DE（MJ/kg） | 3.55 |
| Extruded corn | 27.61 | Crude protein | 19.59 |
| Dehulled soybean meal | 10.33 | Calcium | 0.81 |
| Extruded soybean | 4.50 | Total phosphorus | 0.57 |
| Fish meal | 0.50 | Available phosphorus | 0.37 |
| Whey powder | 8.00 | Lys | 1.36 |
| Soybean protein concentrate | 12.00 | Met+Cys | 0.75 |
| Soybean oil | 1.90 | Thr | 0.79 |
| Sucrose | 3.50 | Trp | 0.23 |
| Limestone | 0.91 |  |  |
| Dicalcium phosphate | 0.74 |  |  |
| Nacl | 0.25 |  |  |
| *L-*Lys-HCl (78%) | 0.38 |  |  |
| *DL*-Met (99%) | 0.17 |  |  |
| Trp (98%) | 0.05 |  |  |
| Thr (98.5%) | 0.02 |  |  |
| Chloride choline | 0.10 |  |  |
| Vitamin premix^1^ | 0.05 |  |  |
| Mineral premix^2^ | 0.20 |  |  |
| Total | 100.00 |  |  |

^1^The premix provides following per kg diet：VA 5512 IU, VD_3_2250 IU, VE 24 mg, VK_3_ 3 mg, VB_2_ 6 mg, VB_6_ 3 mg, VB_12_ 24 μg, folic acid 1.2 mg, nicotinic acid 14 mg, biotin 150 μg,*D*-pantothenic acid 15 mg.

^2^The premix provides following per kg diet：Fe 100 mg, Cu 6 mg, Mn 4 mg, Zn100 mg, I 0.14 mg, Se 0.3 mg

Table S4 Primer sequences and annealing temperature

| Target gene | | Forward primer 5’-3’ | Reverse primer 5’-3’ | | Product length | | Accession number | |  |
| --- | --- | --- | --- | --- | --- | --- | --- | --- | --- |
| EGF | ATCTCAGGAATGGGAGTCAACC | | | TCACTGGAGGATGGAATACAGC | | 165 | | NM_214020.1 | |
| GLP-2 | ACTCACAGGGCACGTTTACCA | | | AGGTCCCTTCAGCATGTCTCT | | 149 | | NM_005671883.1 | |
| ANG4 | ACCACTTGTACGCACTCAGG | | | ACTCATCGAAGTGGACAGGC | | 118 | | NM_001163409.1 | |
| IGF-1 | CTGAGGAGGCTGGAGATGTACT | | | CCTGAACTCCCTCTACTTGTGTTC | | 137 | | NM_001097417.1 | |
| IGF-1R | TTCGCCAGATCCTAGGGGAG | | | TCCCAGCTTTGATGGTCAGG | | 120 | | NM_214172.1 | |
| Occludin | CAGGTGCACCCTCCAGATTG | | | GGACTTTCAAGAGGCCTGGAT | | 110 | | [NM_001163647.2](http://www.ncbi.nlm.nih.gov/entrez/viewer.fcgi?db=nucleotide&id=402746997) | |
| ZO-1 | CTGAGGGAATTGGGCAGGAA | | | TCACCAAAGGACTCAGCAGG | | 105 | | [XM_013993251.1](http://www.ncbi.nlm.nih.gov/entrez/viewer.fcgi?db=nucleotide&id=927096856) | |
| REG3γ | GGCTTGGAACCAAATGCTGG | | | TAGCCAGGGTATGAGCTGGT | | 101 | | XM_005662419.1 | |
| MUC1 | GTGCCGCTGCCCACAACCTG | | | AGCCGGGTACCCCAGACCCA | | 141 | | [XM_001926883.5](http://www.ncbi.nlm.nih.gov/entrez/viewer.fcgi?db=nucleotide&id=927121447) | |
| MUC2 | GGTCATGCTGGAGCTGGACAGT | | | TGCCTCCTCGGGGTCGTCAC | | 181 | | [XM_013989745.1](http://www.ncbi.nlm.nih.gov/entrez/viewer.fcgi?db=nucleotide&id=927100665) | |
| IL1β | ACGTGCAATGATGACTTTGTCTG | | | AGAGCCTTCAGCATGTGTGG | | 113 | | [NM_214055.1](http://www.ncbi.nlm.nih.gov/entrez/viewer.fcgi?db=nucleotide&id=47522925) | |
| IL10 | GACGTAATGCCGAAGGCAGA | | | TGCTCTTGTTTTCACAGGGC | | 133 | | [NM_214041.1](http://www.ncbi.nlm.nih.gov/entrez/viewer.fcgi?db=nucleotide&id=47524185) | |
| β-actin | TCTGGCACCACACCTTCT | | | TGATCTGGGTCATCTTCTCAC | | 114 | | DQ178122 | |

Table S5 Primes and probes for real time PCR of bacteria

| Items | Primer/probe name and sequence(5'-3') | Product length/bp |
| --- | --- | --- |
| *Escherichia coli* | DC-F,CATGCCGCGTGTATGAAGAA |  |
|  | DC-R,CGGGTAACGTCAATGAGCAAA | 96 |
|  | DC-P,(FMA)AGGTATTAACTTTACTCCCTTCCTC(BHQ-1) |  |
| *Lactobacillus* | RS-F,GAGGCAGCAGTAGGGAATCTTC |  |
|  | RS-R,CAACAGTTACTCTGACACCCGTTCTTC | 126 |
|  | RS-P,(FMA)AAGAAGGGTTTCGGCTCGTAAAACTCTGTT(BHQ-1) | |
| *Bifidobacterium* | SQ-F,CGCGTCCGGTGTGAAAG |  |
|  | SQ-R,CTTCCCGATATCTACACATTCCA | 121 |
|  | SQ-P, (FMA) ATTCCACCGTTACACCGGGAA(BHQ-1) |  |
| *Bacillus* | YB-F,GCAACGAGCGCAACCCTTGA |  |
|  | YB-R,TCATCCCCACCTTCCTCCGGT | 92 |
|  | YB-P, (FMA)CGGTTTGTCACCGGCAGTCACCT(BHQ-1) |  |
| Total bacteria | Eub338F,ACTCCTACGGGAGGCAGCAG |  |
|  | Eub518R,ATTACCGCGGCTGCTGG | 200 |
